# Progress of Sanitary Legislation

**Published:** 1855-09

**Authors:** 


### HYGIENIC JURISPRUDENCE.

### PROGRESS OF SANITARY LEGISLATION.

### ACTS.

*Nuisances Removal Act for England,* 1855.—The nuisances included in the act are premises, pools, ditches, gutters, watercourses, privies, urinals, cesspools, drains, ashpits, animals, accumulations, and deposits, in such state, or so kept as to be nuisances, or injurious to health; accumulations and deposits, *bonâ fide* necessities for any business, excepted. The local authorities to carry out the act are local boards of health, councils, bodies of trustees or commissioners, highway boards, committees annually chosen by vestries, boards of inspectors for lighting and watching, and guardians and overseers of the poor, and surveyors of highways—that is, either of these, where those named previous to it do not exist. In extra-parochial places, committees annually elected by the householders. The act also contains provisions for defraying the expenses of its execution. Clause 9 authorises the appointment and payment of sanitary inspectors. Clause 10 provides for notice of nuisances, which may be given by any person aggrieved thereby, any paid officer under the local authority, two or more inhabitant householders, the relieving officer, and officer of the police force, and in case of a model lodging-house, any person appointed for the inspection of such. Clause 11 provides power of entry. Clause 12 provides that in case of nuisance the local authority shall cause a complaint to be made before a justice of the peace, who shall summons the responsible party before two justices, when, if proved, an order for its abatement or discontinuance and for costs shall be made. Clause 14 provides a penalty of ten shillings per day where all due diligence to carry out the order is not made, and of twenty shillings per day where it is wilfully disobeyed, in which latter case the local authority may remove the nuisance at the cost of the person on whom the order is made. Clause 16 provides for appeal against the order, when structural works are required, and no liability to penalty until the appeal is determined, or ceases to be prosecuted. Clause 17 provides, that when a responsible party for a nuisance cannot be found, it may be removed by the local authority. Clause 18 provides for the sale of anything removed by a local authority. Clause 21 provides for the cleansing of ditches, &c., adjoining any highway, and paying damages to those injured thereby. Clause 22 provides for the construction of sewers where necessary. Clause 23 provides that no water shall be fouled by gas washings, &c.; the penalty in case of wilfulness being £200. Clause 25 provides an additional penalty of £20 per day for the continuance of such fouling of water after twenty-four hours notice has been given. Clause 26 provides for the inspection of food, its destruction or disposal when unwholesome, and a penalty of £10 for every piece of meat, parcel of fruit, &c., found exposed for sale when unfit for food. Clause 27 provides that in case of nuisance arising from business or manufacture, the local authority shall, upon the certificate of any medical officer, or two legally qualified medical practitioners, cause the person responsible to be summoned, and be liable to a penalty. This clause is not applicable to any place without the limits of any city, town, or populous district. Clause 29 provides that on the certificate of the medical officer of health, or of two qualified practitioners, as to the overcrowding of any house where the inhabitants consist of more than one family, steps shall be taken for abating it, the person permitting it forfeiting a sum not exceeding forty shillings. The remaining clauses provide penalties for obstructing the execution of the act, for appeals to the quarter sessions, and other procedures.

*Metropolis Local Management Act.*—Sir Benjamin Hall's new Act for the better local management of the metropolis embraces several points connected with sanitary legislation. The Act is framed on a comprehensive basis, and is an able State document. It cannot fail to do good, but whether its full influence will soon be felt depends solely upon the bodies to whom its practical working will be entrusted, viz., the Vestries, the District Boards of Works, and the Metropolitan Board of Works.

The following are in a few words the chief provisions of the Act bearing on sanitary measures. Clause 68 vests either in vestries or district boards of works, all sewers except the mains, which are at present vested in the Metropolitan Commission of Sewers. Clause 72 provides that vestries and district boards shall construct and cleanse sewers, so as they shall not be a nuisance or injurious to health. Clause 73, that in cases of insufficient drainage, the construction of adequate drains may be enforced under penalty. Clause 74, that blocks of houses, in advantageous cases may be drained in combination. Clause 75, that houses shall not be built or rebuilt without drainage and water supply satisfactory to the surveyor of the vestry or district board. Clause 76, that seven days notice shall be given in writing of, in any case of building, rebuilding, or drain making, in default of which such works may be demolished or caused to be altered, and the expenses recovered from their owner. Clause 81, that no house shall be built or rebuilt without suitably fitted with water-closets, under penalty, not exceeding £20. Clause 82 provides for the inspection of drains and similar works. Clauses 86 and 87, for the cleansing and covering of open ditches, etc., and for the compensation of persons prejudicially affected thereby. Clause 88, for the construction of public conveniences. Clauses 98, 99, and 100, for the paving of streets, courts, etc.; likewise for the draining of courts. Clauses 101 and 102 provide for the supervision of the construction of vaults under streets, and for their repairing. Clauses 103 and 104 provide conditions as to the occupation and inspection of underground rooms used as dwellings. Clause 116 for the watering of streets, and gratuitous supply of water by pumps. Clauses 117 and 118 for the cleansing of footways, both by the occupier and by crossing sweepers paid by the parish. Clauses 125, 126, 127 and 128 provide for the appointment of scavengers, for penalty in case of obstruction in the performance of their duties, and for the removal and disposal of the refuse collected. Clause 132 provides for the appointment of legally qualified "medical officers of health" to ascertain the existence of diseases, especially epidemics, to point out local causes of disease, modes of checking and preventing disease, methods of ventilation, etc. Clauses 133 and 134 provide respectively, inspectors of nuisances, and authorities for the execution of the Act. The remaining clauses, referring to the duties and powers of Metropolitan Board of Works, to provisions for defraying expenses, to audit of accounts, reports, appeals, and kindred subjects, do not require further detail. The Act comes into general operation on January 1, 1856.

*Vaccination Bill.*—A bill to provide for the vaccination of the people in England and Wales, to which we beg to call the attention of our readers, and especially of the medical portion of them, has been prepared by the Small-pox and Vaccination Committee of the Epidemiological Society, to carry out the suggestions contained in their memorial to the Board of Health, printed by the House of Commons, March 1st, 1855, and was presented to Sir George Grey and Sir B. Hall by a deputation of the society, headed by the president, Dr. Babington, on the 11th of June last.

The principal alterations comprised in the bill consist in placing the control of public vaccination in the hands of the Board of Health, under the immediate direction of a medical superintendent: the giving power to the Board to appoint any legally qualified medical practitioner a public vaccinator: the extension of the compulsory provisions of the former act, so as to insure the complete vaccination of the people: the abolition of the cumbrous system of vaccination certificates, and the substitution of a proper registration: the securing more adequate remuneration for the public vaccinators.

These are important changes, and, being of an administrative character, can only properly be carried out by the government, as in all probability they will be during the next session of parliament. Meanwhile, the government having informed the committee that the state of public business would not allow their taking the matter up in the past session, the committee accepted gladly an offer made by Mr. Brady to introduce the measure for the sake of having it printed, that its provisions might be made generally known to the profession, and suggestions invited for its improvement before being again presented to the government at the commencement of next session.

### REPORTS.

*Report of the Committee for Scientific Inquiries of the Medical Council of the General Board of Health, in relation to the Cholera Epidemic of* 1854.—Amongst other points the Committee reports respecting the influence of density of population on cholera, from which we learn that density has no decisive influence. Elevation, however, presents a different result, statistics showing that the effects of cholera have been more nearly inverse to the elevation of the soil than proportionate to any other general influence that could be measured. Age also has a striking influence, the danger of an attack varying with age. The mortality as to sex is, males 47, females 45, in 10,000. Mr. Glaisher's meteorological report draws attention to the undue height of the barometer at the worst moments of the epidemic, the influence of heat, the small range of temperature of London to fog, mist and haze, and the predominance of calm; his leading results being that periods of cholera have had their marked meteorological characters, and that these are of such kind as to prevail with increased development in those low London levels where cholera has always most cruelly pressed. Special examinations of the atmosphere by Dr. Thomson and Mr. Rainey present little more than negative interest. Nine sanitary inspectors, appointed to visit all severely affected localities, are unanimous in stating that wherever cholera had become localised, they found connected with it obvious removable causes. Examinations of water by Drs. Thomson and Hassall show that the London water-supply is derived from impure sources. The supply from wells is generally not superior. As to the effects of water, there is no reason to ascribe any appreciable influence to its mineral ingredients, nor to its living animal and vegetable forms. There is, however, no hesitation in speaking of decomposing organic matter as having probably exercised great influence on the spread of cholera. Meteorological observations accord well with the view that the channel of cholera poison is the lungs. Further search for foreign elements possibly entering by respiration presents only a negative result. The Committee state with reference to the exclusive theory as to the stomach being the only channel through which infection can occur, that they find nothing in its support, and that the geographical distribution of cholera expresses, beyond reasonable doubt, its dependence on other than dietetic influences. With reference to communication of the disease from person to person, no large body of evidence, positive or negative, has been received. The report concludes by suggesting for future inquiries, the channel, the personal origin, the period of incubation, and the communication of the disease; also the influence of manufactures and diets on it, consecutive fever, the identity of cholera and diarrhœa, the blood in cholera, obstruction of the capillary circulation, the non-discharge of bile, and the rise of temperature after death.

*Adulteration of Food.*—The first report of the select committee on adulteration of food, etc., with the minutes of evidence, has been issued; from which we find that Drs. Hassall, Normandy, R. D. Thomson, Messrs. Redwood, Warington, Simon, Mitchell, and Blackwell, and Sir J. Gordon, have been examined. General evidence on foods, drinks, water, drugs, and remedial measures, was given by Drs. Hassall and Normandy. Evidence on adulteration of foods was also given by Mr. Mitchell; of tea and drugs, by Mr. Warington; of flour, butter, milk, drugs, and remedies, by Sir. J. Gordon (Mayor of Cork); of flour, coffee, tea, sugar, and water, by Dr. Thomson; on pickles, by Mr. Blackwell (of a pickle-manufacturing firm); on drugs, by Dr. Redwood. Appendices, principally on teas, and diagrams, illustrative of impurities of various waters, accompany the report.

*Report on the Laws and Ordonnances in force in France for the regulation of noxious Trades and Occupations, by Dr. Waller Lewis.* 1855. *(Presented to both Houses of Parliament.)*—This report treats of a great variety of subjects, amongst which we may mention the classification of unwholesome occupations, their effects on health; public abattoirs, and the abattoir system in Paris; the abattoir at Aubervilliers; the manufacture of gut, animal charcoal, bone-boiling, artificial manure, glue, size, leather, candles, Prussian blue, soap-boiling, tallow-melting, buttons, fans, wool, tobacco, sulphate of quinine, cotton, white lead, shot, brick-making, gilding by mercury, gas, brass, percussion caps, fireworks, bronze figures, iron, matches, shoemaking, tailors, etc. We hope to give a complete analysis of Dr. Lewis' useful report in a future number.

---

### RETURNS.

*Chicory.*—From a return to the House of Commons we ascertain that on the 3rd of August 1852, *(Treasury Minute, July 27th)*, the general order of 31st of August 1840, directing no objection to the mixture of chicory with coffee, was rescinded, and mixture not allowed. On the 28th of February 1853, however, this order was rescinded, and mixture again allowed, provided the package containing it was labelled "Mixture of Chicory and Coffee", which label was altered on the 13th of May to "This is sold as a Mixture of Chicory and Coffee". We also learn that 43 Geo. III, c. 129, s. 5, provides that adulterations of, or substitutions for, coffee and cocoa be seized, and the manufacturer, dealer, or seller, forfeit £100. The expenses incurred in carrying out the treasury minutes of 27th July 1852, and 25th of Feb. 1853, have been £2170 : 10 : 5; but the fines of convicted persons have more than covered these expenses.

---

### GENERAL NOTES.

Sir Benjamin's Hall's Temporary Public Health Bill received the Royal Assent on August 14th.

The Lord Advocate's "Burial Grounds (Scotland)" Bill received the Royal Assent on July 23, 1855; Mr. Baines' "Burial of Poor Persons" Bill, on July 30; and Mr. Dunlop's Dwelling Houses (Scotland)" Bill on Aug. 14. Sir W. Somerville's "Dwelling for Labouring Classes (Ireland)" Bill is referred. Sir J. Young's "Intramural Burials (Ireland)" Bill was read the third time on March 23. Viscount Ebrington's "Sewers (House Drainage)" Bill received the Royal Assent on June 15.

*Exposure of Bodies in Churches in Spain.*—The following order has been issued to the governors of the provinces of Spain:

Nothing is more prejudicial to public health than the exposure of dead bodies in churches. All who have treated of public hygiene have insisted on the very great importance, as a sanitary measure, of prohibiting bodies from being brought into places of public worship. The decomposition after death gives rise to noxious emanations, which, being inhaled by the congregation, produce most severe diseases. The correctness of this observation has been recognised in all ages. In 1801, Charles IV. promulgated a decree forbidding the exposure of the body during funerals; and, although prejudices and pride caused the decree to sink into oblivion, there was a renewed attempt to establish good sanitary measures, on September 20, 1849. From an inadequate enforcement of the responsibility imposed by this royal order on the governors, who sanctioned a practice likely to increase by abuse, the custom of exposing bodies during funerals has gone on increasing. The practice mentioned is at all times hurtful; but most especially so in the present sanitary state of the country, and considering the influence which the sight of a dead body has on the mind. It is inconceivably absurd, that although fumigations and disinfectants of all kinds are employed to purify the dwelling where a case of epidemic disease has occurred, the bodies of those who have died of the epidemic are allowed to be carried to the churches, places in general ill ventilated, especially when the number of persons who assemble in them is taken into account. Her Majesty the Queen, recognising the justice of the remarks offered, and the fact that the exposure of the body during funeral is a manifest infraction of royal mandates, has prohibited the act referred to, and makes the governors of the provinces responsible for the least laxity which they may permit. I inform you of this royal order, that it may be punctually and exactly fulfilled. (Signed) Huelves. *Madrid*, 28th August, 1855.

